# Real-time observation of a high-echoic mass in the left ventricle during transcatheter aortic valve implantation: a case report

**DOI:** 10.1093/ehjcr/ytaa392

**Published:** 2020-12-07

**Authors:** Akiko Masumoto, Takeshi Kitai, Mitsuhiko Ota, Kitae Kim, Natsuhiko Ehara, Yutaka Furukawa

**Affiliations:** Department of Cardiovascular Medicine, Kobe City Medical Center General Hospital, 2-1-1 Minatojima-minamimachi, Chuo-ku, Kobe 6500047, Japan

**Keywords:** TAVI, Complications, Stroke, Transoesophageal echocardiography, Case report

## Abstract

**Background:**

Increasing number of symptomatic patients with severe aortic stenosis is treated with transcatheter aortic valve implantation (TAVI). Stroke is one of the most serious complications of TAVI, and the majority of cerebral events in patients undergoing TAVI have an embolic origin.

**Case summary:**

A 90-year-old female underwent trans-femoral TAVI for symptomatic severe aortic stenosis. Just before the implantation of the transcatheter heart valve (THV), transoesophageal echocardiography (TOE) showed a mobile, high-echoic mass attached to the THV, which gradually enlarged to 26 mm, then spontaneously detached from the THV and flowed up the ascending aorta, disappearing from the TOE field of. After the procedure, the patient presented with ischaemic stroke. The patient’s stroke was thought to have resulted from the embolism migrating to the distal cerebral arteries.

**Discussion:**

The detailed images acquired with TOE during TAVI enabled the prompt identification of the unusual intracardiac mass.

**Learning points**

- Detailed images acquired with transoesophageal echocardiography can enable the prompt identification of the unusual intracardiac mass during transcatheter aortic valve implantation (TAVI).
- Pre-procedural planning for TAVI using multimodality imaging is essential to avoid complications including cerebral embolisms.

## Introduction

Increasing numbers of symptomatic patients with severe aortic stenosis are treated with transcatheter aortic valve implantation (TAVI), as it has evolved into a safe and reproducible procedure. One of the most serious complications of TAVI is stroke, which was identified in 10.0% of TAVI patients upon careful neurological examination;^1^^,^^2^ embolism was found to be the major cause of stroke. Transcatheter aortic valve implantation is increasingly performed under local anaesthesia, but in our case, detailed images acquired with transoesophageal echocardiography (TOE) during TAVI enabled prompt identification of unusual intracardiac mass, which eventually embolized to cerebral arteries.

## Timeline

**Table:** 

Before procedure	A 90-year-old patient with dyspnoea on exertion was found to have severe aortic stenosis with a peak trans-valvular jet velocity of 4.1 m/s. Considering her age, frailty and the risk for surgical aortic valve replacement, she was referred for transcatheter aortic valve implantation (TAVI)
0:00	General anaesthesia was initiated for TAVI
00:45	Temporary pacemaker was placed
01:50	First attempt at advancing the expandable sheath from left femoral artery failed, and the wire was exchanged to Lunderquist, without success
02:02	A buddy wire was added, but the expandable sheath did not advance
02:17	Balloon angioplasty was performed at the abdominal aorta, and the sheath advanced
02:29	Heparin added, and activated clotting time (ACT) was 307 s
02:50	A guidewire was placed in the left coronary artery to prevent obstruction
02:54	ACT was 298 s. The balloon aortic valvuloplasty was applied under rapid pacing
03:09	Delivery system was advanced
	Transoesophageal echography (TOE) showed the sudden appearance of a high-echoic mass attached to the valve
03:18	The transcatheter heart valve (THV) was implanted under rapid pacing
03:20	The intracardiac mass grew in length and moved in and out of the left ventricle
03:29	The mass detached from the THV and flowed up the ascending aorta, disappearing from the TOE screen
03:40	ACT was 347 s
04:07	Cerebral angiography was performed, and no major cerebral trunk was occluded
04:34	The sheaths were extracted, and the procedure was over
Post- procedure	The patient presented with hemianopsia and ataxia. Acute cerebral infarction was found in magnetic resonance imaging. After rehabilitation, the patient was able to walk independently with score 3 on the modified Rankins scale

## Case presentation

A 90-year-old female was referred for dyspnoea on exertion. On auscultation, mid-systolic grade III murmur was heard at the left sternal border. Electrocardiogram showed sinus rhythm, and her left ventricular ejection fraction was preserved (66%). Transthoracic echocardiography showed severe aortic stenosis with a peak trans-valvular jet velocity of 4.1 m/s, a mean pressure gradient of 42 mmHg, and an aortic valve area of 0.75 cm^2^. The degree of aortic regurgitation was trivial, with no other structural heart abnormality. She had a history of hypertension and hypothyroidism. The patient had been taking administered azosemide 7.5 mg, amlodipine 5 mg, candesartan 8 mg, and cilnidipine 5 mg. She was mildly frail with frailty index of 5 and Katz index was 5 out of 6. N-terminal prohormone of brain natriuretic peptide was 212 pg/mL (normal value <450 pg/mL for patients aged 75–99 years). Estimated mortality risk for isolated surgical aortic valve replacement was 5.28% according to the Society of Thoracic Surgery risk score. Considering her age, frailty, and the intermediate risk for surgical aortic valve replacement, trans-femoral TAVI for symptomatic severe aortic stenosis was planned by the heart team. Coronary angiography showed severe stenosis in the proximal right coronary artery that was fixed by drug-eluting stent implantation before TAVI in staged approach and no significant stenosis in left coronary artery.

**Figure 1:**
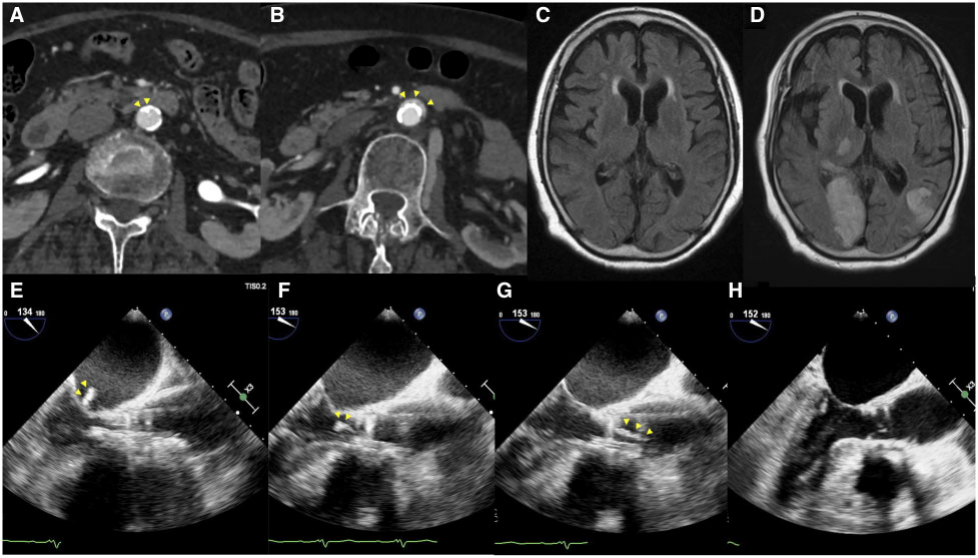
(*A*) Abdominal computed tomography prior to transcatheter aortic valve implantation. A 5 mm calcified plaque can be seen in the lumen of the abdominal aorta (arrowhead). (*B*) Abdominal computed tomography after transcatheter aortic valve implantation. Localized aortic dissection can be seen (arrowhead). (*C*) Cerebral magnetic resonance imaging prior to transcatheter aortic valve implantation. (*D*) Cerebral magnetic resonance imaging after transcatheter aortic valve implantation. Acute cerebral infarction was found on magnetic resonance imaging after transcatheter aortic valve implantation. The patient presented with hemianopsia and ataxia. (*E–H*) Transoesophageal echocardiography during transcatheter aortic valve implantation; mid-oesophageal long-axis views. A high echoic, 10 mm long mass (arrowhead) is attached to the transcatheter heart valve (*E*). Subsequently, the mass is moving in and out of the left atrium and the left ventricle (*F*). After implantation, the mass gradually enlarged to a length of 26 mm (*G*). Then, the mass spontaneously detached from the transcatheter heart valve and flowed up the ascending aorta, disappearing from the transoesophageal echocardiography field of view (*H*).

Before the procedure, computed tomography (CT) scan showed a 5 mm width calcified plaque protruding into the lumen of the abdominal aorta (*Figure 1A*). Since the lumen measured 10.0 mm, and the 14 Fr expandable sheath was 6.0 mm, we determined that the 14 Fr expandable sheath could pass through the abdominal aorta. The basal annulus area was 343 mm^2^, and the perimeter was 66.3 mm. Trans-femoral-TAVI was planned with an access from the left femoral artery with a 23 mm balloon-expandable valve.

Because the ostium of the left coronary artery was low, at 8 mm above the annulus, a guidewire was placed in the beginning of the procedure to prevent coronary obstruction. The height of right coronary artery origin was sufficient at 11 mm. To confirm that the transcatheter heart valve (THV) was not occluding the left coronary artery during the procedure, general anaesthesia was given to enable the real-time observation with TOE. A temporary pacemaker was placed in the right ventricle through right jugular vein.

During the procedure, the 14 Fr expandable sheath did not initially pass the abdominal aorta. The extra-stiff wire was exchanged for a Lunderquist extra-stiff wire, and another Lunderquist extra-stiff wire was added from the right femoral artery as a buddy wire, but the sheath could not still ascend farther than the terminal aorta. Balloon angioplasty was carefully performed at the abdominal aorta with a 12 mm balloon at 4 atm, which finally enabled the sheath to ascend past the calcified plaque. Heparin was given and activated clotting time (ACT) was 307 s.

A 5 Fr Amplatz left 1 catheter and a straight wire were used to cross the aortic valve, and then exchanged to a 5 Fr pigtail catheter and an angled hydrophilic wire. Finally, the wire was exchanged with a Safari extra small wire in the left ventricle. After ACT was reconfirmed at 298 s, the balloon aortic valvuloplasty was applied with a 20 mm balloon. Afterwards, the delivering system was advanced. Just before the implantation of the THV, TOE showed the sudden appearance of a high echoic, 10 mm long mass attached to the THV (*Figure 1E*). We discussed whether the mass could possibly embolize, but the cerebral embolic protection (CEP) device was not available in Japan. Moreover, the delivering system had already advanced past the native aortic valve, and as retraction of the device would cause the mass to detach from the THV, we decided to proceed with the implantation. After the implantation of the THV under rapid right ventricular pacing, the mass gradually enlarged to 26 mm, which then spontaneously detached from the THV and flowed up the ascending aorta, disappearing from the TOE field of view (*Figure 1E–H* and *Video 1*). The stroke team immediately performed cerebral angiography, but no major cerebral trunk was occluded.

After the procedure, the patient presented with hemianopsia and ataxia. Acute cerebral infarction was found in magnetic resonance imaging that was not observed pre-procedurally (*Figure 1C and D*). The patient’s stroke was thought to have resulted from the embolism migrating to the distal cerebral arteries. There were no signs of systemic embolization in CT.

## Discussion

TAVI is increasingly performed under local anaesthesia without TOE, but more detailed images can be acquired with TOE, for prompt identification of unusual intracardiac masses. Here, we used general anaesthesia and TOE to ensure the THV was not obstructing the left coronary artery, which was located low in our patient. The majority of cerebral events in patients undergoing TAVI have an embolic origin that includes thrombi, calcific and atherosclerotic material, leaflet tissue, and calcific deposits from the native valve.^3^^,^^4^

Many high-intensity transient signals are detected on transcranial Doppler during the stage of valve implantation, suggesting release of calcific debris and valve tissue from the native aortic valve during release of the prosthesis.^5^ The 14 Fr expandable sheath has an outer dimension of 6 mm, but its dimension increased to the maximum of 7.1 mm during advancement of the THV.^6^ Here, one explanation to the origin of the mass is that the intima of the abdominal aorta might have been damaged by the sheath insertion and the balloon inflation, and subsequently the torn intima might have been carried to the left ventricle with the delivering device, which then was observed with TOE as a high-echoic mass. The following elongation of the mass could partially have been due to thrombus formation on the surface of the torn intima and debris, although anticoagulation was maintained at an appropriate level. Moreover, since the mass seemed to have appeared after the balloon aortic valvuloplasty, it could have originated from the leaflet tissue of the native aortic valve. All three of the native aortic leaflets were degenerated with dense calcification, and it could partially have been torn at the valvuloplasty. Pre-procedural CT showed no left ventricular outflow tract calcification.

Some studies have investigated the effects of the CEP devices during the procedure. In a propensity-matched analysis of the clinical studies of the CEP in TAVI, use of the dual filter CEP was associated with a significantly lower rate of peri-procedural stroke compared with unprotected TAVI (1.88% vs. 5.44%, odds ratio 0.35, 95% confidence interval 0.17–0.72; *P* = 0.0028).^7^ However, because the analysis was possibly driven by a single-centre non-randomized study, it is still inconclusive whether CEP provides clinical benefit in reducing peri-procedural stroke.

In conclusion, the detailed images acquired with TOE during TAVI enabled the prompt identification of the unusual intracardiac mass.

### Patient perspective

After rehabilitation for acute cerebral infarction, the patient walked independently with score 3 on the modified Rankins scale. Computed tomography showed localized abdominal aortic dissection at the site of calcified plaque (*Figure 1B*). At 1 year after the procedure, the patient was alive and was in New York Heart Association I.

## Lead author biography

**Figure:**
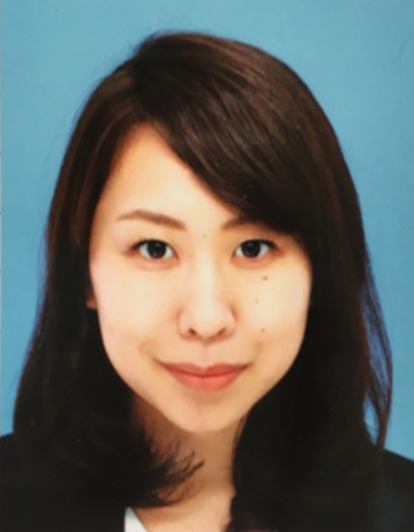


Akiko Masumoto achieved her licence to practice medicine at Osaka University, Japan. After completing junior residency at Kobe City Medical Center General Hospital in Kobe, she is currently a senior resident of the Department of Cardiovascular Medicine at Kobe City Medical Center General Hospital. Her interest is in structural heart diseases and intensive cardiac care.

## Supplementary material

Supplementary material is available at *European Heart Journal - Case Reports* online.

**Slide sets:** A fully edited slide set detailing this case and suitable for local presentation is available online as Supplementary data.

**Consent:** The author/s confirm that written consent for submission and publication of this case report including image(s) and associated text has been obtained from the patient in line with COPE guidance.

**Conflict of interest:** none declared.

**Funding:** None declared.

## Supplementary Material

ytaa392_Supplementary_Data
