# Supplementary material for: Real-time observation of a high-echoic mass in the left ventricle during transcatheter aortic valve implantation: a case report
Source: Eur Heart J Case Rep. 2020 Dec 7;4(6):1–4. doi: 10.1093/ehjcr/ytaa392 (PMC7891275; doi:10.1093/ehjcr/ytaa392)
Supplement: ytaa392_Supplementary_Data [file ytaa392_supplementary_data.zip › ytaa392-suppl_data/Slide-Set3.pptx]

## Slide 1
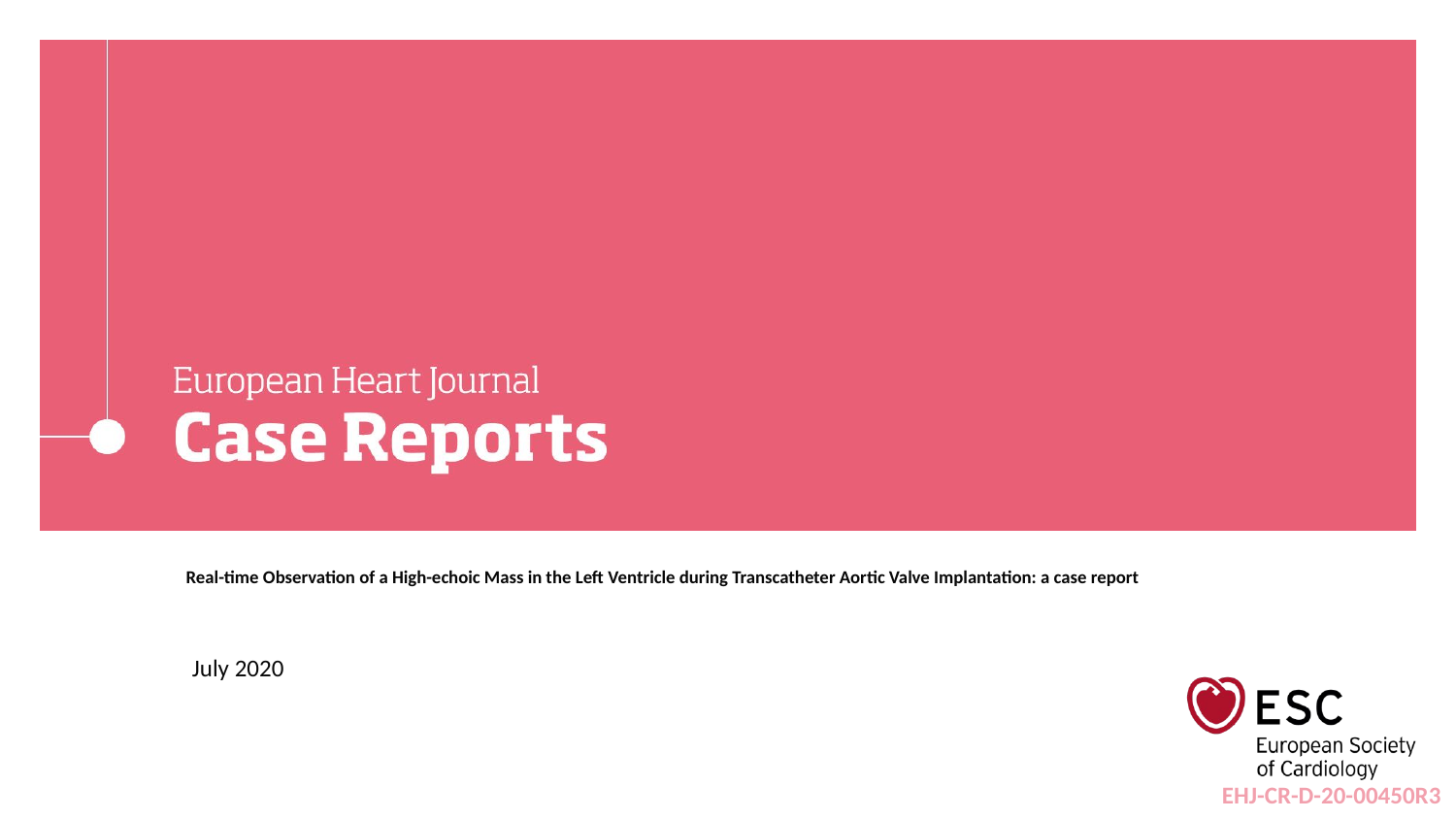

# Real-time Observation of a High-echoic Mass in the Left Ventricle during Transcatheter Aortic Valve Implantation: a case report
July 2020
EHJ-CR-D-20-00450R3

## Slide 2
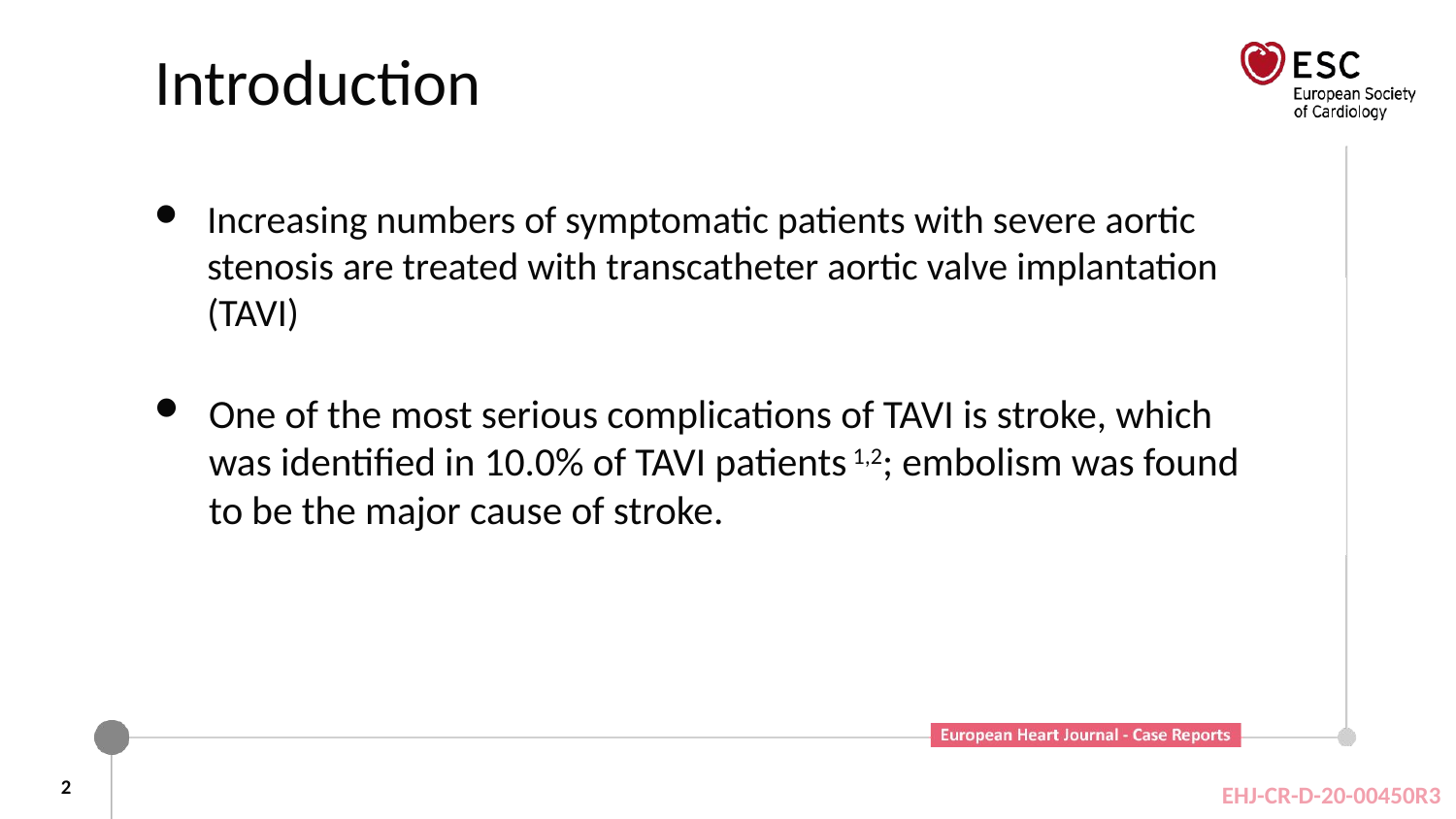

# Introduction
Increasing numbers of symptomatic patients with severe aortic stenosis are treated with transcatheter aortic valve implantation (TAVI)
One of the most serious complications of TAVI is stroke, which was identified in 10.0% of TAVI patients 1,2; embolism was found to be the major cause of stroke.
2
EHJ-CR-D-20-00450R3

## Slide 3
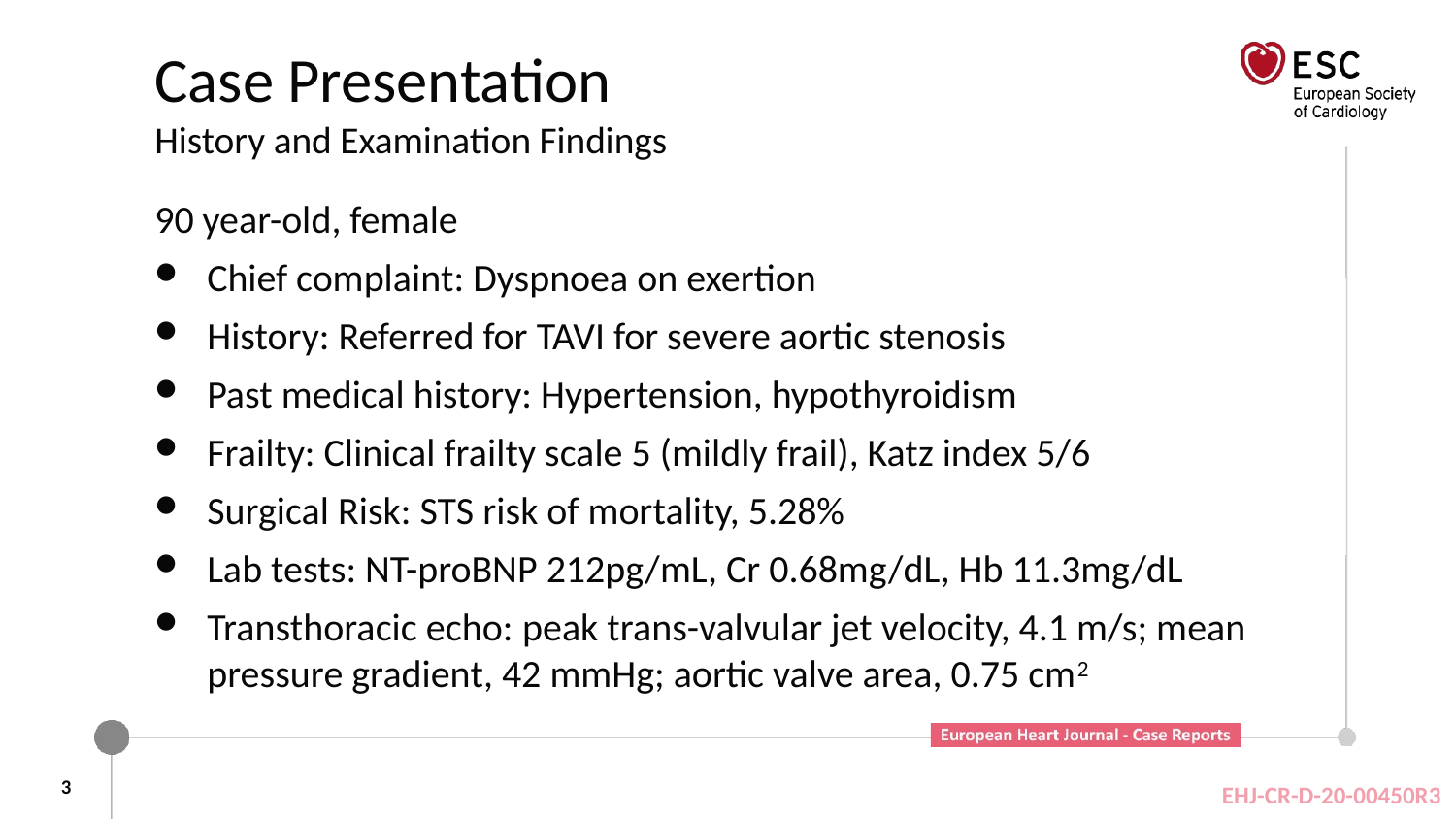

# Case Presentation History and Examination Findings
90 year-old, female
Chief complaint: Dyspnoea on exertion
History: Referred for TAVI for severe aortic stenosis
Past medical history: Hypertension, hypothyroidism
Frailty: Clinical frailty scale 5 (mildly frail), Katz index 5/6
Surgical Risk: STS risk of mortality, 5.28%
Lab tests: NT-proBNP 212pg/mL, Cr 0.68mg/dL, Hb 11.3mg/dL
Transthoracic echo: peak trans-valvular jet velocity, 4.1 m/s; mean pressure gradient, 42 mmHg; aortic valve area, 0.75 cm2
3
EHJ-CR-D-20-00450R3

## Slide 4
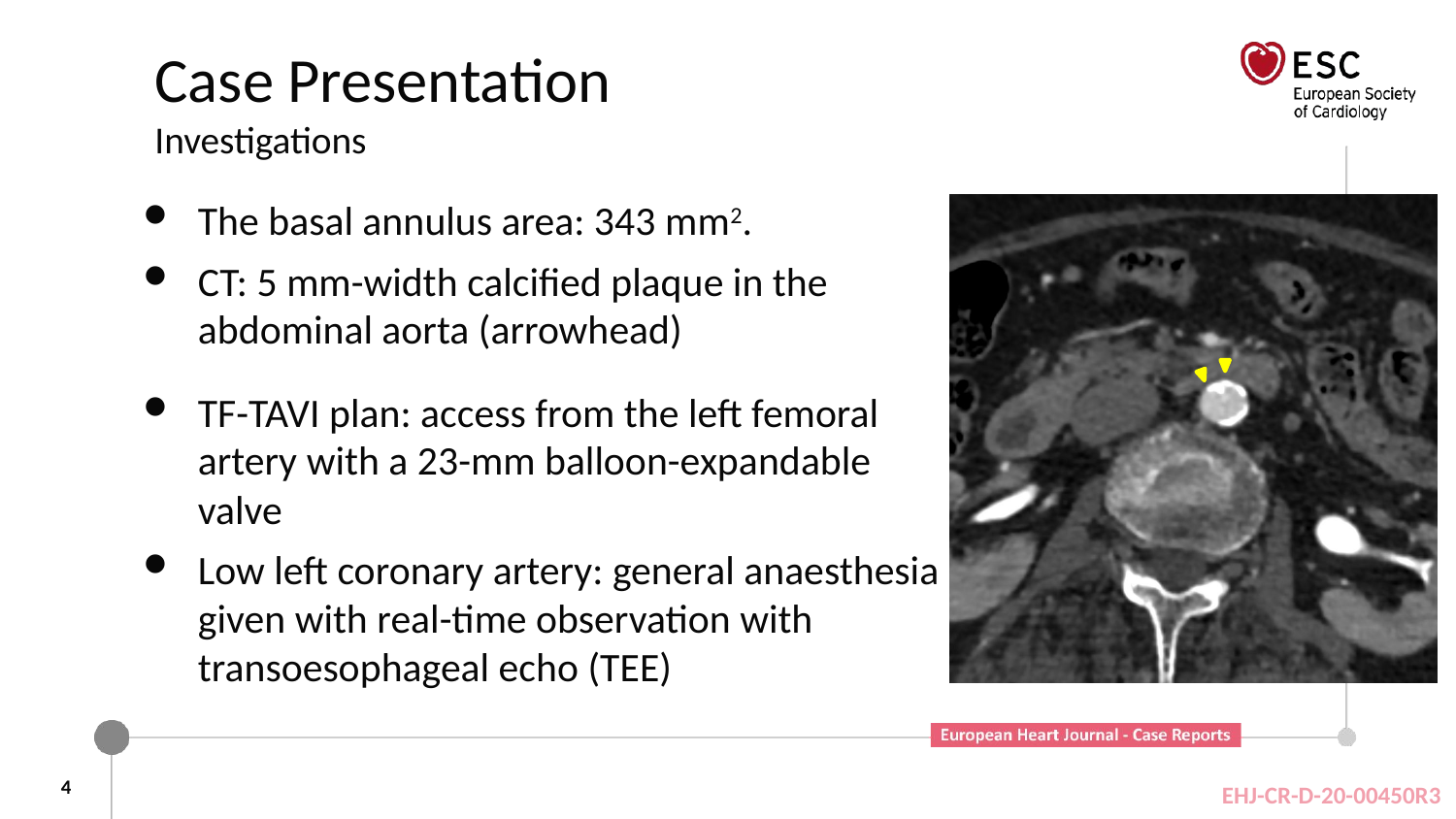

# Case Presentation Investigations
The basal annulus area: 343 mm2.
CT: 5 mm-width calcified plaque in the abdominal aorta (arrowhead)
TF-TAVI plan: access from the left femoral artery with a 23-mm balloon-expandable valve
Low left coronary artery: general anaesthesia given with real-time observation with transoesophageal echo (TEE)
4
EHJ-CR-D-20-00450R3

## Slide 5
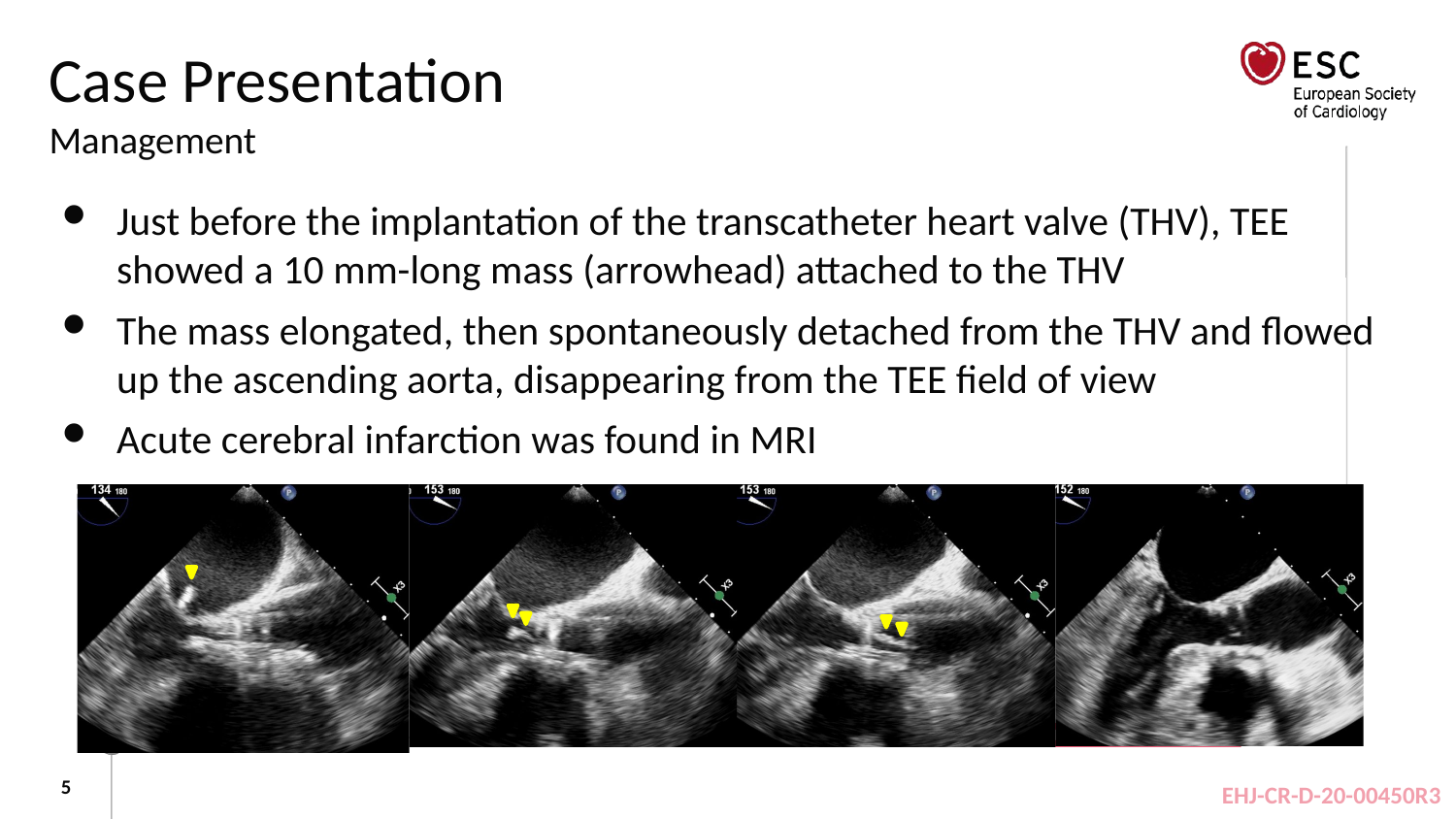

# Case Presentation Management
Just before the implantation of the transcatheter heart valve (THV), TEE showed a 10 mm-long mass (arrowhead) attached to the THV
The mass elongated, then spontaneously detached from the THV and flowed up the ascending aorta, disappearing from the TEE field of view
Acute cerebral infarction was found in MRI
5
EHJ-CR-D-20-00450R3

## Slide 6
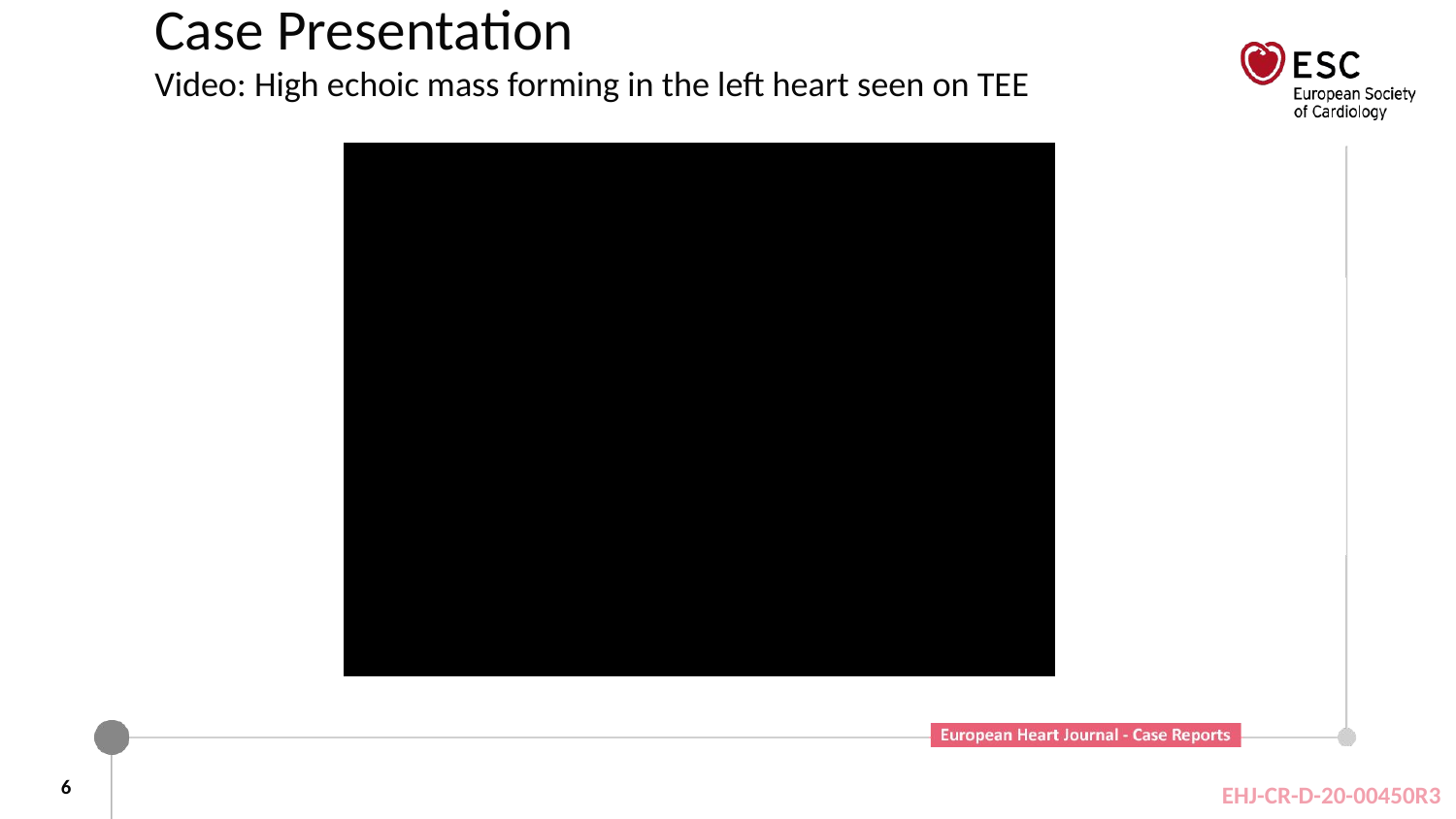

# Case Presentation Video: High echoic mass forming in the left heart seen on TEE
6
EHJ-CR-D-20-00450R3

## Slide 7
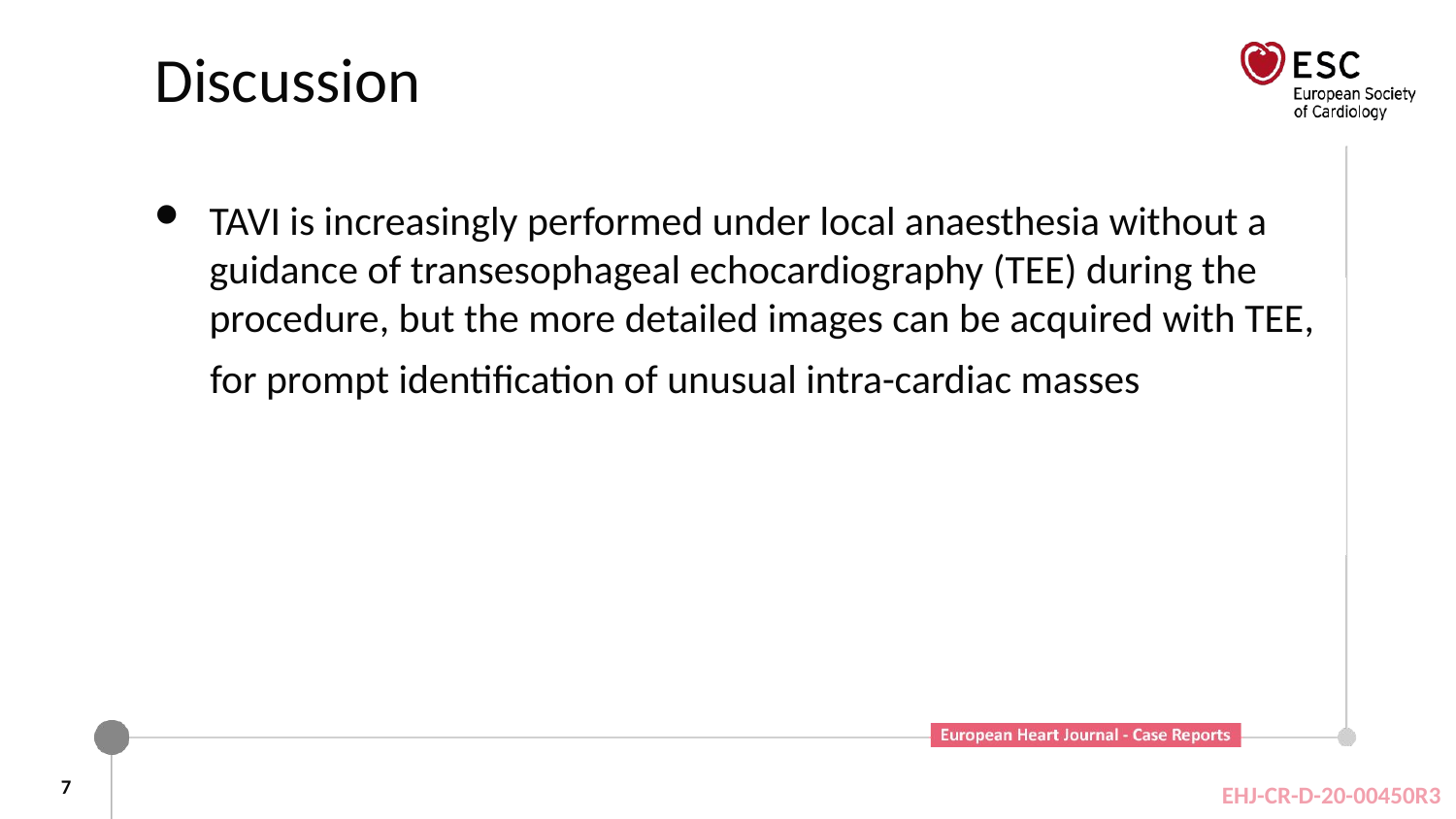

# Discussion
TAVI is increasingly performed under local anaesthesia without a guidance of transesophageal echocardiography (TEE) during the procedure, but the more detailed images can be acquired with TEE,
 for prompt identification of unusual intra-cardiac masses
7
EHJ-CR-D-20-00450R3

## Slide 8
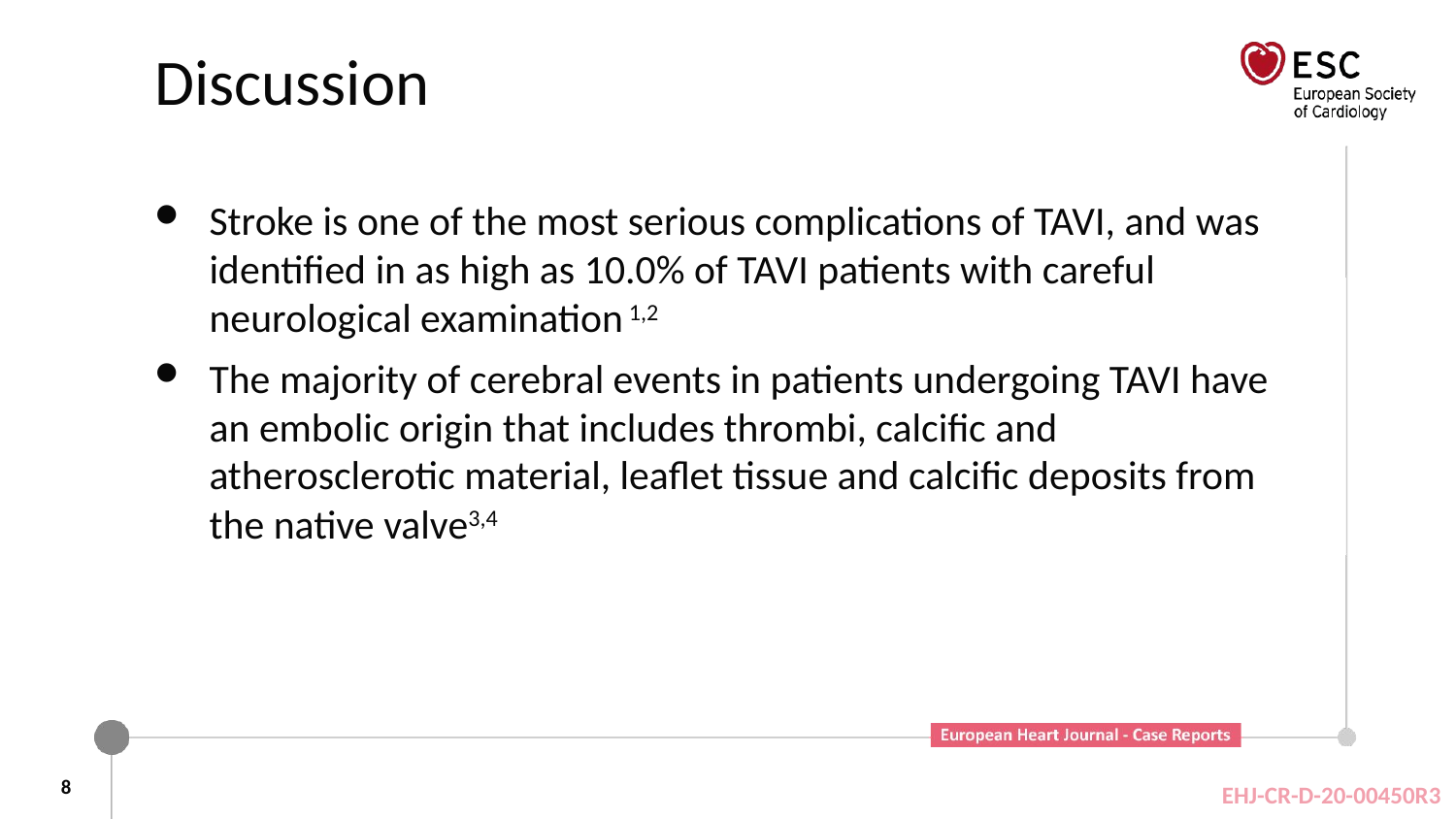

# Discussion
Stroke is one of the most serious complications of TAVI, and was identified in as high as 10.0% of TAVI patients with careful neurological examination 1,2
The majority of cerebral events in patients undergoing TAVI have an embolic origin that includes thrombi, calcific and atherosclerotic material, leaflet tissue and calcific deposits from the native valve3,4
8
EHJ-CR-D-20-00450R3

## Slide 9
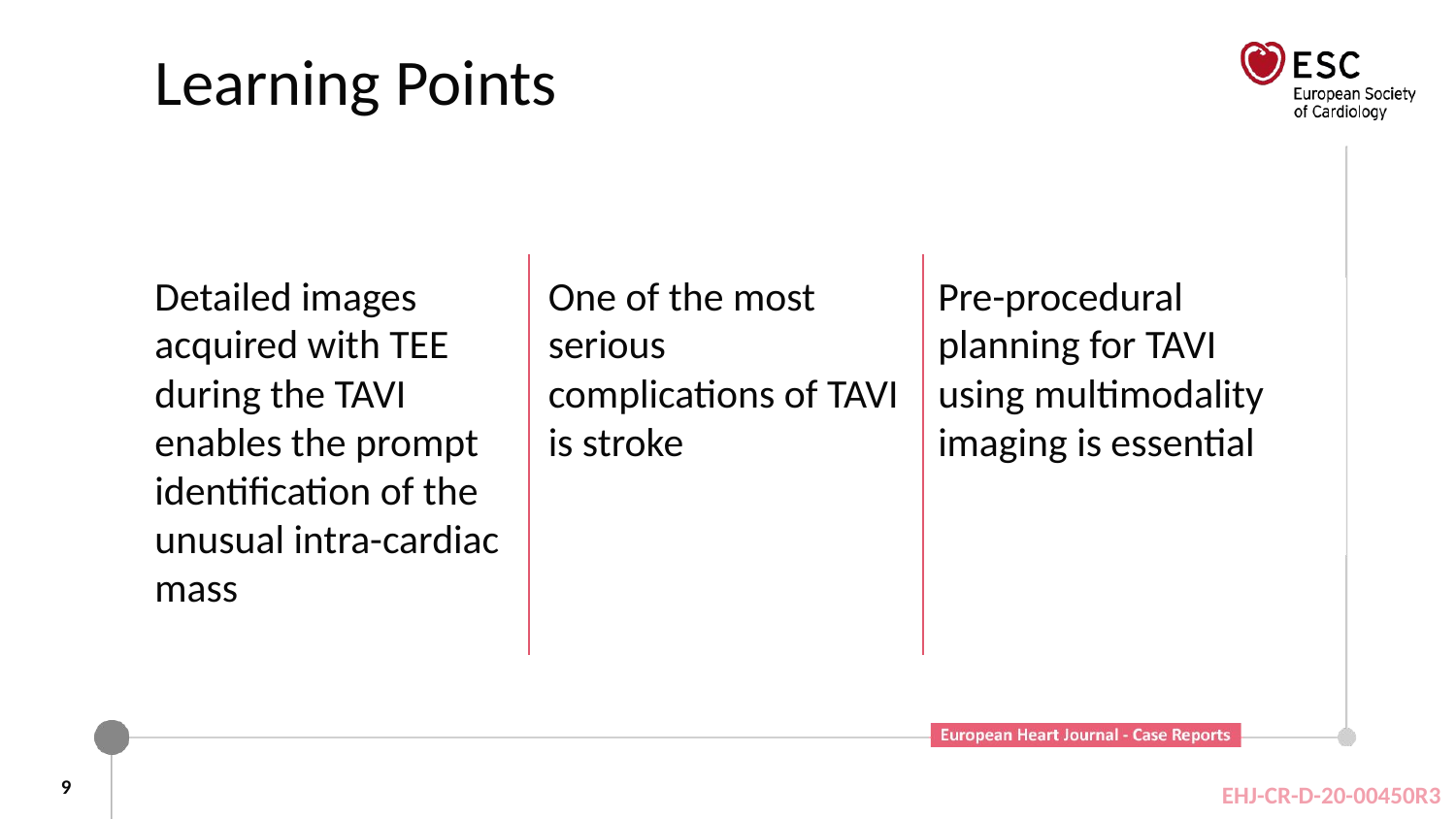

# Learning Points
Detailed images acquired with TEE during the TAVI enables the prompt identification of the unusual intra-cardiac mass
One of the most serious complications of TAVI is stroke
Pre-procedural planning for TAVI using multimodality imaging is essential
9
EHJ-CR-D-20-00450R3

## Slide 10
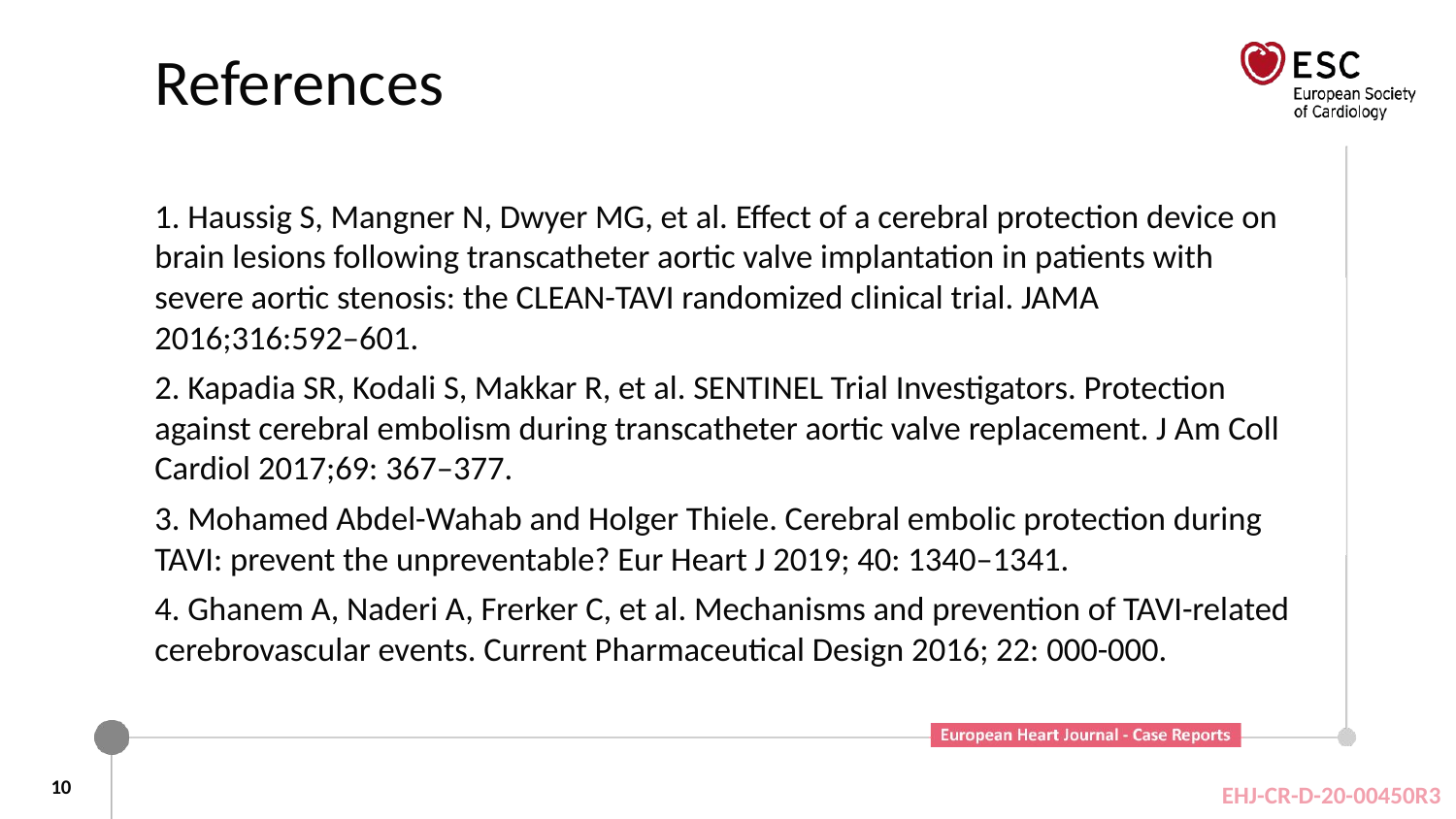

# References
1. Haussig S, Mangner N, Dwyer MG, et al. Effect of a cerebral protection device on brain lesions following transcatheter aortic valve implantation in patients with severe aortic stenosis: the CLEAN-TAVI randomized clinical trial. JAMA 2016;316:592–601.
2. Kapadia SR, Kodali S, Makkar R, et al. SENTINEL Trial Investigators. Protection against cerebral embolism during transcatheter aortic valve replacement. J Am Coll Cardiol 2017;69: 367–377.
3. Mohamed Abdel-Wahab and Holger Thiele. Cerebral embolic protection during TAVI: prevent the unpreventable? Eur Heart J 2019; 40: 1340–1341.
4. Ghanem A, Naderi A, Frerker C, et al. Mechanisms and prevention of TAVI-related cerebrovascular events. Current Pharmaceutical Design 2016; 22: 000-000.
10
EHJ-CR-D-20-00450R3
